# Response of Two Different Wheat Varieties to Glow and Afterglow Oxygen Plasma

**DOI:** 10.3390/plants10081728

**Published:** 2021-08-20

**Authors:** Pia Starič, Silva Grobelnik Mlakar, Ita Junkar

**Affiliations:** 1 Jožef Stefan Institute, Jamova Cesta 39, 1000 Ljubljana, Slovenia; ita.junkar@ijs.si; 2 Faculty of Agriculture and Life Sciences, University of Maribor, Pivola 10, 2311 Hoče, Slovenia; silva.grobelnik@um.si

**Keywords:** cold plasma, nonthermal plasma, wheat, plants, afterglow plasma, glow plasma, SEM, roots, vigor, germination

## Abstract

Cold plasma technology has received significant attention in agriculture due to its effect on the seeds and plants of important cultivars, such as wheat. Due to climate change, wherein increasing temperatures and droughts are frequent, it is important to consider novel approaches to agricultural production. As increased dormancy levels in wheat are correlated with high temperatures and drought, improving the germination and root growth of wheat seeds could offer new possibilities for seed sowing. The main objective of this study was to evaluate the influence of direct (glow) and indirect (afterglow) radio-frequency (RF) oxygen plasma treatments on the germination of two winter wheat varieties: Apache and Bezostaya 1. The influence of plasma treatment on seed surface morphology was studied using scanning electron microscopy, and it was observed that direct plasma treatment resulted in a high etching and nanostructuring of the seed surface. The effect of plasma treatment on germination was evaluated by measuring the germination rate, counting the number of roots and the length of the root system, and the fresh weight of seedlings. The results of this study indicate that the response of seeds to direct and indirect plasma treatment may be variety-dependent, as differences between the two wheat varieties were observed.

## 1. Introduction

Cold plasma technology has a wide range of biological applications, of which decontamination, sterilization, improved seed germination, plant growth, and improved resistance to abiotic stress are of high interest for its application in the agricultural industry [1,2,3,4,5,6,7,8]. Plasma agriculture is a relatively new field of research that offers great potential for various agricultural applications. Plasma treatment techniques could offer an alternative way to treat seeds, which could significantly improve germination as well as reduce or even eliminate the use of environmentally unfriendly chemicals. To control plant diseases and overcome unfavorable growth conditions, developing new technologies to improve the resistance of crops to diseases and various abiotic stresses with a minimal negative impact on the environment is essential. Wheat is one of the most predominant crops, historically and presently, and is economically essential worldwide [9]. Numerous research articles have investigated the effects of cold plasma treatment on improved wheat seed germination, growth, changes in metabolism, and stress resistance [7,8,10,11,12,13,14]. However, the results of these studies are not always consistent and many conflicting results can be found in the literature. The main reason for this is not only the use of different seed varieties, but also the use of different plasma systems, which operate under specific conditions that are usually not well-described. Thus, it is very hard to compare results from different studies. In addition, only a limited number of studies dealt with the effects of different plasma species (ions, atoms, excited species, vacuum ultraviolet radiation, etc.) on the seed surface, which may hold the key to understanding the complex interaction mechanisms taking place between the seed surface and plasma. It is usually difficult to eliminate contributions from different plasma species; however, in the afterglow regime, only neutral molecules and atoms are present. Thus, our work aimed to study the effects of different plasma regimes on two different wheat varieties. Two types of seeds were exposed to glow (direct) and afterglow (indirect) plasma to study changes in the seed surface and their influence on germination.

A few authors have investigated how cold plasma treatment affects different seed varieties of various crops. Volin et al. studied the influence of cold plasma treatment on two pea seed varieties. Their studies revealed no significant differences in the final germination rate between both varieties in response to cold plasma treatment [15]. On the other hand, Ling et al. found that the two oilseed rape cultivars responded differently to the plasma seed treatment. One variety was drought-sensitive (Zhongshuang 7), and the other (Zhongshuang 11) was drought-tolerant. The Zhongshuang 7 variety demonstrated a better germination rate after the plasma treatment compared to untreated seeds. On the other hand, Zhongshuang 11 did not show improved seed germination after the plasma treatment compared to the control. However, both varieties showed greater root and shoot length after the plasma treatment of the seeds [5]. Yodpitak et al. conducted a study on six rice cultivars. Their experiments showed an increase in germination rate, root length, and shoot length in all rice varieties after the plasma treatment of the seeds compared to the control, but the degree of improvement was variety-dependent. Scanning electron microscopy (SEM) analysis showed that the plasma treatment of seeds resulted in a significant etching effect of the seed surface. The seed coat appeared smoother after plasma treatment compared to the rough seed coat of untreated seeds [16]. Similar findings of cold plasma effects on seed coat have also been reported by other researchers [4,8,17,18]. 

Although several authors have conducted extensive research on the cold plasma treatment of seeds, to the best of our knowledge, none of them have investigated how different wheat varieties respond to plasma treatment, or how glow and afterglow plasma treatment affects seed germination and growth. Previously mentioned studies have investigated the variety-dependent changes in other plant species such as rice, oilseed rape, and pea. Since wheat is an important crop, the objective of our study was to evaluate how two different winter wheat varieties (*Triticum aestivum* L. Apache and Bezostaya 1) respond to radio-frequency (RF) oxygen plasma treatment in two different plasma regimes, the glow and afterglow regime. The effects of plasma treatment on the seeds were evaluated by analyzing the germination rate, root length and the number of roots, shoot height, the fresh weight of seedlings, and seed surface morphology. Our study indicates that the influence of plasma treatment might be variety-dependent, as statistically significant effects were observed in the number of roots, the length of the total root system, and the fresh weight of seedlings.

## 2. Results

### 2.1. Glow and Afterglow Plasma Treatment Caused Morphological Changes on the Seed Surface

**Figure 1:**
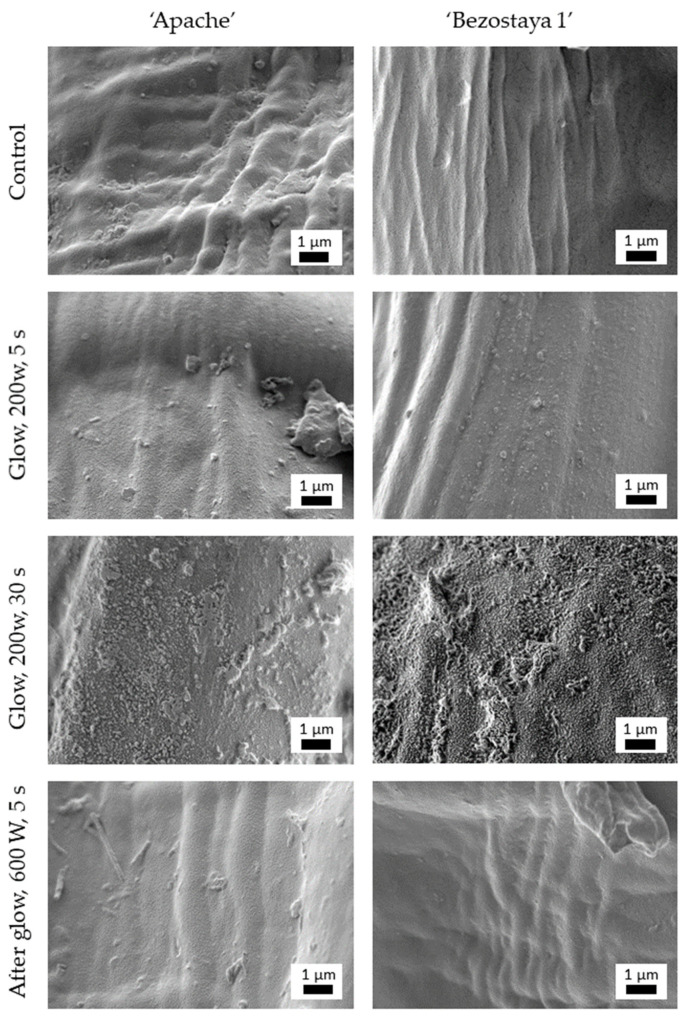
SEM images of the wheat seed coat of the Apache and Bezostaya 1 varieties of non-treated seeds, seeds treated with the glow regime (direct plasma) for 5 and 30 s at a power of 200 W, and seeds treated with the afterglow regime (indirect plasma) for 5 s and power of 600 W.

The main difference between the exposure of seeds to glow and afterglow regimes lies in the interaction of the plasma species with the seed surface [19]. In the afterglow regime, the ions from the plasma are recombined on the wall of the plasma reactor before reaching the seed surface; this results in lower etching and reduces the thermal heating caused by ion bombardment compared to the glow plasma regime [20,21]. Our studies of seed morphology determined from SEM analysis show that the afterglow regime results in much lower etching effects, as virtually no changes in surface morphology were observed. The untreated seed coat exhibited rough net-like structures, while nanostructured morphologies were observed after the exposure of seeds to the plasma glow regime (Figure 1). After 5 s of treatment at 200 W, the surface morphology of the seed was altered and visible remnants of etched debris were observed. The removal of the seed surface coat, presumably a preferential etching of waxes, was even more evident after 30 s of treatment. Longer exposures caused greater etching, resulting in a structured nano-rough seed surface. However, treatment in the afterglow regime at a much higher power input did not change the seed surface morphology compared to the control. Differences between the two wheat varieties, Apache and Bezostaya 1, were also observed. It is already known that the etching rate is highly dependent on the chemical structure of the surface, as the counterparts that are easily etched are removed from the surface much faster, causing nanostructuring. The more pronounced nanostructured surface was observed in Bezostaya 1 after 30 s of treatment (Figure 1), indicating differences in seed coat composition between these two varieties. 

### 2.2. Germination Process

**Figure 2:**
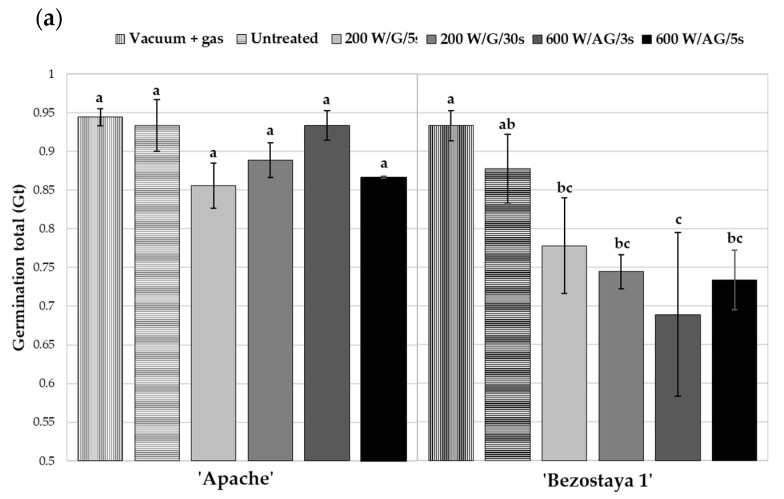

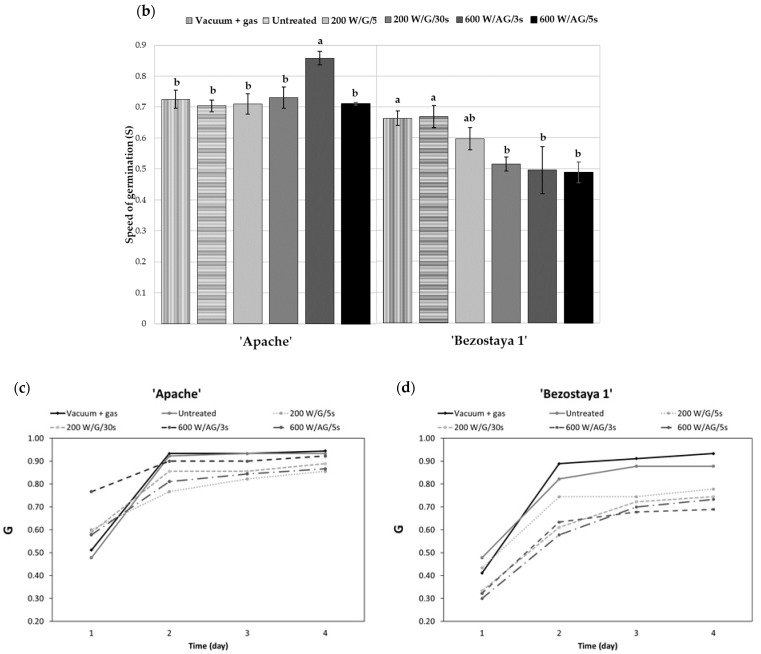
The germination total (**a**), speed of germination (**b**), and accumulated germination in time (**c**,**d**) of two wheat varieties depending on seed treatment with oxygen plasma at different treatment conditions. “G” in (**c**,**d**) represents the proportion of germinated seeds. The displayed values are the mean ± SEM of three replications. The different letters (a–c) indicate statistically significant differences among treatments according to Duncan’s test (α = 0.05).

As reported by Anjum and Bajwa [22], the calculated parameter S (speed of germination) describes the germination process more accurately than Gt (germination rate) alone. This is also confirmed in our study where oxygen plasma treatment significantly influenced parameter S in both wheat varieties (Figure 2), while no statistically significant effect on the Gt parameter was observed in Apache (Figure 2a). The parameter S (Figure 2b) in Apache is the highest after seed treatment in the afterglow regime at an input power of 600 W for 3 s and is significantly higher compared to the control. The control samples were untreated seeds and seeds exposed to vacuum conditions. No significant differences were observed between these two samples. Interestingly, plasma treatment decreased all germination parameters in Bezostaya 1. The most notable decrease in Gt was observed in seeds exposed to afterglow plasma at 600 W for 3 s, while the negative effect on the parameter describing germination speed was also shown when seeds were exposed to glow plasma at 200 W for a longer time (30 s). These results indicate that the two wheat varieties respond differently to the same plasma treatment conditions. No significant changes in Gt were observed in Apache; however, a notable increase in germination speed (S) was observed in seeds exposed to glow plasma at 600 W for 3 s. On the other hand, seeds from Bezostaya 1 treated with glow and afterglow plasma showed a statistically significant negative trend: a lower germination rate and slower germination than the control. Regarding the plasma treatment conditions, it seems that glow (200 W for 30 s) and afterglow (600 W for 5 s) plasma treatments have similar effects on the Gt and S parameters in seeds of this variety; in both treatment regimes, the Gt and S parameters were similar. Thus, it could be speculated that both treatment conditions have a similar influence on germination and the speed of growth for a particular variety. From the application point of view, the plasma treatment conditions could be optimized to achieve the desired effect in the shortest time possible. The accumulated germination in time of the Apache seeds treated with afterglow plasma for 3 s exhibited faster seed germination on the first day compared to the control and other plasma treatments (Figure 2c). This result supports the statistically significant faster germination (S) in the same plasma treatment. On the other hand, Bezostaya 1 exhibited lower final germination (Gt) and slower germination in all plasma treated seeds except the treatment in glow plasma for 5 s, which is also shown in the accumulated germination in time (Figure 2d).

### 2.3. Morphological Characteristics of Seedlings

The morphological parameters measured on the germinating wheat plants show that the variety (V) had a statistically significant effect on root number (Rn) and total root system length (Rt). In contrast, individual root length (Ri) and the length of plumule (Pl) were not influenced by factor V (Table 1). Furthermore, the root-to-shoot ratio (R/S) (in fresh weight) was not affected by either V or plasma treatment (T). 

**Figure 3:**
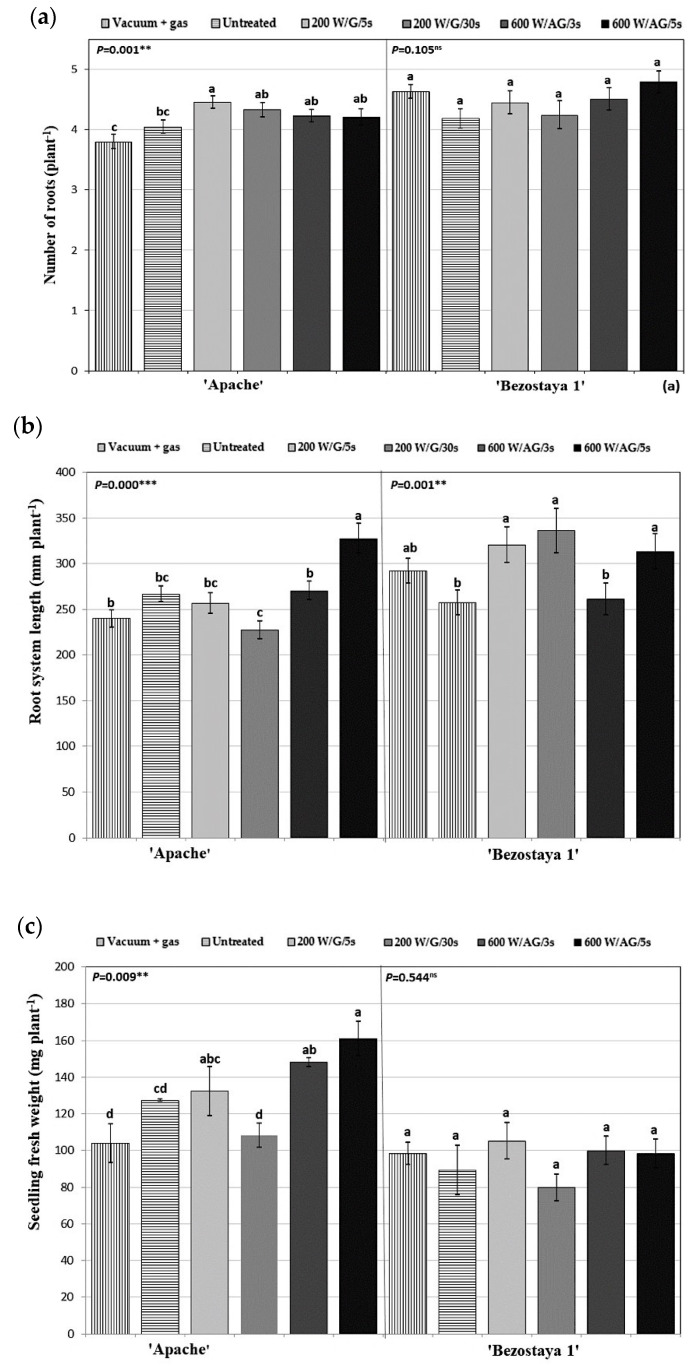

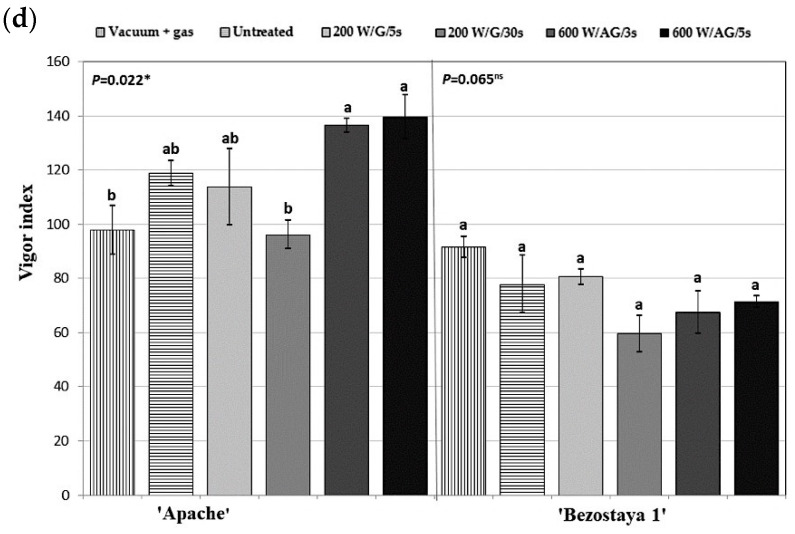
The number of roots per plant (**a**), root system length (**b**), seedling fresh weight (**c**), and seed vigor (**d**) of two wheat varieties depending on seed treatment with oxygen plasma at different treatment conditions. The displayed values are the mean ± SEM of three replications. The different letters (a–c) indicate statistically significant differences among treatments according to Duncan’s test (α = 0.05). Non-significant differences are marked as “ns”.

Neither the glow nor afterglow plasma treatment of Bezostaya 1 had a statistically significant effect on the number of roots per plant. However, the plasma treatment of Apache seeds exhibited a statistically significant increase in the number of roots after both the glow and afterglow plasma treatments compared to the vacuum control (Figure 3a). However, compared to the control samples, only the glow plasma treatment at 200 W for 5 s increased the root number. The root system length (Figure 3b) of the plasma treated seeds of Bezostaya 1 showed no statistically significant difference compared to the vacuum control. However, the root systems of both glow plasma treated samples, and the samples treated for 5 s with 600 W afterglow plasma, had statistically significantly longer root systems compared to the control. The Apache seeds treated with glow plasma at 200 W for 30 s, on the other hand, showed a statistically significant decrease in root system length compared to the vacuum control, but no significance compared to the control. The treatment of Apache seeds in an afterglow plasma regime at 600 W for 5 s resulted in an increase in the root system length compared to the vacuum control and control samples. In terms of root system length and the number of roots per plant, Apache and Bezostaya 1 responded differently to cold plasma treatment. This could be important, as a large and deep root system may be an advantage when plants grow in moist soil, while a shallower root system may be more beneficial when frequent rainfall during the growing season is the primary source of moisture for the crop plants [23].

The fresh weight of the Bezostaya 1 seedlings of cold-plasma-treated seeds did not show statistically significant differences compared to the control or vacuum control (Figure 3c). On the other hand, the Apache seedlings showed an increase in fresh weight when treated with the glow regime (5 s at 200 W), as well as with both afterglow treatment conditions. However, compared to the controls, only the afterglow plasma treatment of seeds (600 W/AG/3 s and 600 W/AG/5 s) exhibited an increase in the fresh weight of the seedlings.

Seed vigor describes the ability of seeds to germinate and establish seedlings rapidly, uniformly, and robustly under various environmental conditions. It is not a single measurable characteristic, but a concept associated with aspects of viable seed performance (e.g., the rate and uniformity of seed germination and parameters describing seedling growth). Seed vigor can describe a complex interaction between seed genetic and environmental components, with genetics being a less understood component of such interactions in the context of producing a robust seed [24]. In the case of Bezostaya 1, cold plasma had no statistically significant effect on its seed vigor index (Figure 3d). However, the seeds of Apache showed an increase in their vigor index when treated with afterglow plasma (600 W for 3 and 5 s).

## 3. Discussion

It has been shown that each wheat variety has different coping mechanisms and interactions with highly reactive oxygen plasma species. This may be mainly attributed to the different chemical structures of the seed surface, seed size, and genetic diversity within the species [25,26,27]. The results suggest that Bezostaya 1 is more sensitive to plasma treatment and has poor coping mechanisms for responding to such treatments. In this case, the seed germination rate was lower after plasma treatment, but there were no evident physiological changes in the root system length, the number of roots, or the fresh weight of seedlings following different plasma treatment conditions. Bezostaya 1 was predominantly sown in the 1970s. At that time, it had a high yield potential, but new wheat varieties with higher yields and stress tolerances for various abiotic factors were developed [28]. Apache showed a better responsiveness and adaptability to cold plasma treatment compared to Bezostaya 1. Apache was developed in 1998 and is a newer wheat variety with a higher yield potential compared to Bezostaya 1. Apache also exhibits resistance to water and nitrogen deficits by developing a deep root system under unfavorable conditions [29]. Thus, the ability of each wheat variety to cope with abiotic stress could be a key factor in seed response to cold plasma treatment. By optimizing plasma treatment conditions for specific wheat varieties, the length of the root system can be altered and thus adapted for growth in various environments. 

The cold plasma treatment of wheat seeds (Apache and Bezostaya 1) in a glow plasma regime resulted in the nanostructuring of the seed coat, which was not observed in the afterglow plasma regime. This is mainly due to the presence of ions in the glow regime, which cause strong surface etching. In addition, glow plasma contains a higher concentration of relatively aggressive reactive chemical species with a higher energy as well as vacuum ultraviolet radiation (VUV). In contrast, only longer-lived reactive chemical species with a lower energy are present in afterglow plasma [21]. The thermal heating of the samples in the afterglow region is practically negligible, whereas the glow region of plasma can cause significant sample (seeds) heating due to strong ion bombardment [20,21]. Our study indicates that the afterglow treatment of Apache increases the vigor index, root system, fresh weight of seedlings, and the speed of germination compared to the control. Therefore, the application of the afterglow plasma regime could be of interest to further studies on seed processing and the optimization of plasma treatment conditions.

The experiments in this study were carried out on two genetically different winter wheat varieties, which show different response mechanisms and tolerance to the cold plasma treatment of seeds. The results indicate that the response of seeds and seedlings to cold plasma treatment cannot be generalized to seed species based solely on results obtained for one variety. One plant species can have large genetic variations between different varieties [30], which may play a crucial role in seed response to cold plasma treatment and later seedling growth. However, it is important to emphasize that other factors (not only genetic), such as growth location, soil, and weather conditions, also play an important role in seed development and could have an important impact on how seeds respond to plasma treatment.

Our findings in this study present the first steps toward the question of whether or not plasma treatment is variety-dependent. In this study, a laboratory-scale plasma reactor was used to study the influence of the plasma glow and afterglow region on two different wheat varieties. Only smaller amounts of seeds have been tested, as the plasma reactor only allows the treatment of smaller quantities of seeds. Due to the limited number of samples treated in the laboratory plasma reactor, the results and statistical analysis were based on three biological replicates with a relatively small sample size. However, statistically significant results were obtained for specific parameters, indicating that plasma treatment is wheat-variety-dependent. A future experimental setup that enables seed treatment in glow and afterglow plasma, with a larger number of seeds per treatment, would significantly contribute to current discussions of the interaction of cold plasma treatment with seeds. Plasma reactors currently used in research and seed treatment are mostly laboratory-scale plasma reactors, in which only a limited number of seeds can be treated [4,31,32]. For the future application of plasma technologies in agriculture and to obtain meaningful and statistically well-supported results, the development and use of larger, well-defined plasma reactors are necessary.

## 4. Materials and Methods

### 4.1. Seed Material

The seeds of winter wheat varieties (*Triticum aestivum* L.) Apache and Bezostaya 1 were obtained from a private collection. Apache is one of the leading modern wheat varieties, while Bezostaya 1 is an old, extensive-type wheat variety, which today is important mainly in wheat breeding [25,26,33]. The tested varieties differed in thousand kernel weight (TKW), grain color, and quality. Apache has a TKW of 44–48 g; it is a high-yielding variety of moderate-to-poor bread-making quality (quality group B2/C) [25]. Bezostaya 1, on the other hand, has a lower TKW (41–44 g) and is an old Russian variety with a lower yield, which is still used in wheat breeding programs as a variety with a high bread-making quality and resistance to low temperatures [26,27]. Seeds of both wheat varieties were produced in the same year in the same field (in Krog near Murska Sobota, Slovenia). Both wheat varieties were sown, treated, harvested, and stored under the same conditions.

### 4.2. Characteristics of Plasma and Seed Treatment

**Figure 4:**
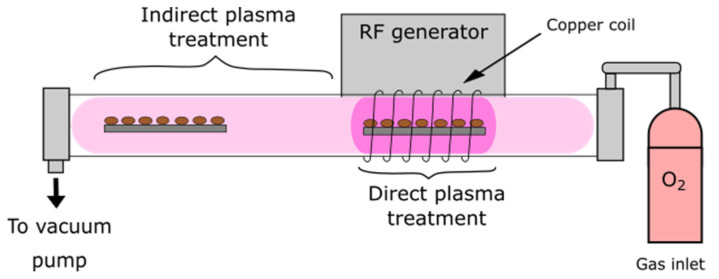
A schematic representation of the plasma system used in the treatment of wheat seeds.

The plasma treatment was carried out using an in-house designed plasma system, illustrated in Figure 4. Four batches of 90 seeds per variety were exposed to oxygen plasma generated by an RF discharge. The pressure was set to 50 Pa and the input power to 200 W. In this case, seeds were treated in the glow region (direct plasma treatment) for 5 and 30 s. The treatment in the afterglow region (indirect treatment) was carried out at an input power of 600 W for 3 and 5 s. The control samples represented untreated seeds and seeds exposed to vacuum conditions, with the same gas flow as during the plasma treatment.

### 4.3. SEM Imaging and Sample Preparation

For SEM analysis, the seeds were mounted onto aluminum stubs using carbon tape and coated with a thin layer of platinum using a Gatan 682 Precision Etching and Coating System (Gatan Inc. Pleasanton, CA, USA). The samples were imaged by a JSM-7600F Schottky field emission SEM (Jeol Ltd., Tokyo, Japan), and the representative images are shown.

### 4.4. Seed Germination, Growth Conditions, and Measurements (Parameters Assessed)

The germination experiments were conducted three hours after the plasma treatment. Seeds from each treatment with controls were divided into three equal replicates (each replicate contained 30 seeds) and evenly placed within sterile Petri dishes (10 cm in diameter) lined with filter paper (No. 1 Whatman International, Maidstone, UK). The filter paper was initially moistened with 5 mL of distilled water, and additionally 3 mL of water was added on the third and fourth day of the experiment. The sealed Petri dishes were arranged in a completely randomized design and incubated at 25 ± 1 °C for four days and an additional three days at 20 ± 1 °C (altering 12 h light/12 h darkness cycle). 

Germination was recorded every 24 h for four days when untreated seeds reached maximum germination, 100.0% in Apache and 97% in Bezostaya 1. A seed was considered to have germinated when the radicle was visible (~1 mm long). In addition to the total germination (*Gt*, final germination proportion) parameter, the speed of germination (*S*) was calculated as recommended by Anjum and Bajwa [22]. Indices *Gt* and *S* were calculated as Equations (1) and (2):

(1) $$Gt=G4=\frac{number\;of\;germinated\;seeds}{30\;seeds}{,}^{\;}$$

(2) $$S=\left(\frac{G1}{1}\right)+\;\left(G2-G1\right)\times \frac{1}{2}\;+\;\left(G3-G2\right)\times \frac{1}{3}\;+\;\left(G4-G3\right)\times \frac{1}{4}{,}^{\;}$$

where *G* is the proportion of germinated seeds obtained during the first (G1) to fourth (G4) day of experiment. The maximum value possible for index *S* is 1.

After seven days of incubation, seedlings were photographed and the lengths of the individual seminal root and shoot were measured with a stereomicroscope (Olympus SZ61, Japan). The fresh weight of the root and shoot were assessed, and the seed vigor index (SV) was calculated according to Equation (3) [27]:

(3) $$\mathrm{SV}=\frac{\mathrm{Fresh}\;\mathrm{weight}\;\mathrm{of}\;\mathrm{seedlings}\;\left(\mathrm{mg}\right)\times \;\mathrm{Gt}}{100}.$$

### 4.5. Statistical Analyses

The obtained results were analyzed using a Statgraphics Centurion XV (Statpoint Technologies, 2005). To improve the normality of distribution and homogeneity of variance (Cochrans C and Bartlett tests), the data describing germination proportion (Gt) was arcsine-transformed. After the multifactor analysis of variance (ANOVA, *p* < 0.05), a one-way ANOVA was carried out to test the effects of plasma treatment within each wheat variety. A comparison of means was performed by Duncan’s test (α = 0.05). Results are presented as an average of three replications with a standard error of mean (±SEM).

## 5. Conclusions

Overall, it can be concluded that not only plasma treatment parameters, but also plant variety and/or seed characteristics, may play a crucial role in optimizing plasma parameters for seed treatment. The physiological responses of plants to the plasma treatment of seeds could be variety-dependent due to different genetic profiles and other factors such as growing location, soil conditions, and the general environmental conditions under which the seeds developed before their plasma treatment. The results of this study serve as a stepping stone for future research in plasma agriculture, as comparisons of varieties of the same plant species could provide important clues for how plasma technology affects seeds and seedlings. It is important to emphasize that results obtained under controlled laboratory conditions may differ from studies conducted in the field where environmental factors are not controlled and could affect plant growth, development, and yield differently. 

For the routine use of cold plasma technology in agriculture, it is important to further investigate the exact mechanisms of how treating seeds with cold plasma affects seed germination and seedling growth in relation to specific plant varieties (genotypes) and plasma treatment conditions. This could provide new insights concerning the complex interaction mechanisms of plasma species with the seed surface and biochemical mechanisms underlying the physiological changes in seedlings and plants. It is crucial to investigate which plasma components contribute to the changes in seed physiology and what mechanisms are triggered in seeds by cold plasma treatment. These findings could enable the agricultural industry to fully utilize the potential of plasma technology in the future.

## Figures and Tables

**Table 1:** The analysis of variance (F-ratio) of chosen morphological parameters (root number, Rn; length of individual root, Ri; root system length, Rt; plumula length, Pl; seedling fresh weight, Fw; root-to-shoot ratio, R/S and seed vigor index, SV).

Source of Variation	Degree of Freedom	Rn	Ri	Rt	Pl	Fw	R/S	SV
Variety (V)	1	13.18 ***	2.47 ns	16.20 ***	1.30 ns	44.25 ***	0.25 ns	98.36 ***
Treatment (T)	5	2.21 ns	6.41 ***	4.73 ***	4.35 ***	4.59 **	0.87 ns	3.40 *
V × T	5	3.24 **	19.92 ***	5.78 ***	2.18 ns	2.32 ns	0.09 ns	5.11 **
Residuals	22	1.42035	1086.79	13,707.6	1030.5	249.69	0.02	163.81

Mean square of residual; ***, **, * Significant at *p* < 0.001, *p* < 0.01 and *p* < 0.05, respectively; ns = not significant.

## Data Availability

The data presented in this study are available in this article.
